# Supplementary material for: Bayesian estimation of community size and overlap from random subsamples
Source: PLoS Comput Biol. 2022 Sep 19;18(9):e1010451. doi: 10.1371/journal.pcbi.1010451 (PMC9522272; doi:10.1371/journal.pcbi.1010451)
Supplement: S1 Text — (PDF) [file pcbi.1010451.s001.pdf]

S1 Text: Factorization of the joint posterior distribution

$$
\begin{aligned}
p(s, R_a, R_b \mid C_a, C_b) &= p(s \mid C_a, C_b, R_a, R_b) \cdot p(R_a, R_b \mid C_a, C_b) && \text{(S1-1)}\\
&= p(s \mid C_a, C_b, R_a, R_b) \cdot p(R_a \mid C_a, C_b) \cdot p(R_b \mid C_a, C_b) && \text{(S1-2)}\\
&= p(s \mid C_a, C_b, R_a, R_b) \cdot p(R_a \mid C_a) \cdot p(R_b \mid C_b) && \text{(S1-3)}\\
&= p(s \mid n_a, n_b, n_{ab}, R_a, R_b) \cdot p(R_a \mid C_a) \cdot p(R_b \mid C_b) && \text{(S1-4)}
\end{aligned}
$$

The first equality is an application of the probability identity $p(A, B) = p(A \mid B)p(B)$. The second equality uses the independence of R_a and R_b. For the third equality, note that the count data for parasite b contains no pertinent information relative to parasite a's repertoire size that parasite a's own count data does not contain. Thus, $p(R_a \mid C_a, C_b) = p(R_a \mid C_a)$ and, similarly, $p(R_b \mid C_a, C_b) = p(R_b \mid C_b)$. The fourth equality is the claim that

$$
p(s \mid C_a, C_b, R_a, R_b) = p(s \mid n_a, n_b, n_{ab}, R_a, R_b) \qquad \text{(S1-5)}
$$

which follows from the fact that the number of times each gene was observed (i.e., the counts) informs the repertoire size as the example above showed. However, when the repertoire sizes are known, only the n_a, n_b, and n_{ab} values from the count data are pertinent to the overlap size.
