# Supplementary material for: Bayesian estimation of community size and overlap from random subsamples
Source: PLoS Comput Biol. 2022 Sep 19;18(9):e1010451. doi: 10.1371/journal.pcbi.1010451 (PMC9522272; doi:10.1371/journal.pcbi.1010451)
Supplement: S2 Text — (PDF) [file pcbi.1010451.s002.pdf]

S2 Text: Theorems enabling efficient computations

Theorem 1. *Let C be the count data resulting from sampling m elements uniformly with replacement from a set with R elements. Let n be the number of unique elements drawn from C. Then, when R is known, we can think of C as a vector*

$$C = (c_1, c_2, \dots, c_n, \underbrace{0, 0, \dots, 0}_{R-n \text{ zeros}})$$

where the c_i correspond to the number of times each sampled element was drawn and we have $\sum_{i=1}^{n} c_i = m$. Let $u = (u_1, u_2, \dots, u_Q)$ be the unique ***nonzero*** *numbers in C and let f_j be the number of times u_j appears in C. Then, the distribution of $C \mid R$ is given by*

$$p(C \mid R) = \frac{R!}{(R-n)! \cdot f_1! \cdot f_2! \cdots f_Q!} \cdot \frac{m!}{c_1! \cdot c_2! \cdots c_n!} \cdot \frac{1}{R^m} \tag{S2-1}$$

Proof. First note that, given R *labeled* elements each with equal probability of being sampled, the multinomial distribution gives the probability of observing any given count data. This is almost the probability that we are interested in except that, for us, the elements are not labeled. That is, as an example, count data $C = (2,3)$ is the same as $C = (3,2)$. So, $p(C \mid R)$ is the multinomial probability multiplied by the number of unique permutations of the counts. The multinomial probability is given by

$$\frac{m!}{\prod_{i=1}^{R} c_i!} \prod_{i=1}^{R} \left(\frac{1}{R}\right)^{c_i} = \frac{m!}{c_1! \cdot c_2! \cdots c_n!} \cdot \left(\frac{1}{R}\right)^{c_1} \left(\frac{1}{R}\right)^{c_2} \cdots \left(\frac{1}{R}\right)^{c_n} \tag{S2-2}$$

$$= \frac{m!}{c_1! \cdot c_2! \cdots c_n!} \cdot \left(\frac{1}{R}\right)^{\sum_{i=1}^{n} c_i} \tag{S2-3}$$

$$= \frac{m!}{c_1! \cdot c_2! \cdots c_n!} \cdot \left(\frac{1}{R}\right)^{m} \tag{S2-4}$$

The number of unique permutations of the counts is the same as the number of R-letter words containing $Q+1$ unique letters, $u_0, u_1, \dots, u_Q$, where letter u_i appears f_i times for $i \neq 0$ and letter u_0 appears $R-n$ times. This number is given by the multinomial coefficient

$$\begin{array}{c}\text{number of unique permutations}\\ \text{of } (c_1,c_2,\dots,c_n,0,0,\dots,0)\end{array} = \frac{R!}{(R-n)! \cdot f_1! \cdot f_2! \cdots f_Q!} \tag{S2-5}$$

And, thus,

$$p(C \mid R) = \frac{R!}{(R-n)! \cdot f_1! \cdot f_2! \cdots f_Q!} \cdot \frac{m!}{c_1! \cdot c_2! \cdots c_n!} \left(\frac{1}{R}\right)^{m} \tag{S2-6}$$

■

Theorem 2. *Let C be the count data resulting from sampling m elements uniformly with replacement from a set with R elements. The count data C consists of the unique elements sampled and the number of times each element was sampled. Let n be the number of unique elements sampled and let $p(R)$ be the prior distribution on the (unknown) set size R. Then, for fixed C and m,*

$$p(R \mid C) = p(R \mid n)$$

That is, $p(R \mid C)$ depends only on the unique number of elements sampled n and not the number of times each element was sampled.

Proof. First note it is impossible for the set size R to be less than number of unique elements sampled n. So, when $R < n$, $p(R \mid C) = 0$.

For $R \geq n$, we can think of the fixed count data C as a vector

$$C = (c_1, c_2, \ldots, c_n)$$

where n is the number of elements sampled and the c_i are the number of times each sampled element was sampled. From Theorem 1, we know that

$$p(R \mid C) \propto p(C \mid R) \cdot p(R) = \frac{R!}{(R-n)! \cdot f_1! \cdots f_Q!} \cdot \frac{m!}{c_1! \cdot c_2! \cdots c_n!} \left(\frac{1}{R}\right)^m \cdot p(R) \tag{S2-7}$$

where $u_1, u_2, \ldots, u_Q$ are the unique numbers *other than zero* in C and f_i is the number of times u_i appears in C. Dropping all the terms that do not depend on R gives

$$p(R \mid C) \propto \frac{R!}{(R-n)!} \cdot \frac{1}{R^m} \cdot p(R) \tag{S2-8}$$

We now turn to $p(R \mid n)$. First, using Bayes' rule and ignoring the denominator term that does not depend on R, we have

$$p(R \mid n) \propto p(n \mid R) \cdot p(R) \tag{S2-9}$$

$p(n \mid R)$ is the probability that n unique elements were sampled from a set with R elements after m uniform draws with replacement. This probability amounts to the distribution of the number of unique items in a multinomial sample, in which the multinomial probabilities are equal. Derived elsewhere (e.g., see [39], §3),

$$P(n \mid R) = \frac{R!}{(R-n)!} \frac{1}{R^m} \left\{ m \atop n \right\} \tag{S2-10}$$

where $\left\{ m \atop n \right\}$ is a Stirling number of the second kind.

Plugging this result into $p(R \mid n)$ and dropping the Stirling number term which does not depend on R gives

$$p(R \mid n) \propto p(n \mid R) \cdot p(R) \tag{S2-11}$$

$$\propto \frac{R!}{(R-n)!} \cdot \frac{1}{R^m} \cdot p(R) \tag{S2-12}$$

which, as a function of R, is the same expression we found for $p(R \mid C)$. Thus, for fixed count data C,

$$p(R \mid C) = p(R \mid n) \tag{S2-13}$$

■

In the context of estimating *var* repertoire sizes and assuming PCR samples *var*s uniformly, this result means that only knowing the sampling effort m and the number of unique *var*s sampled n is as informative as knowing all the counts per gene.
