# Supplementary material for: Bayesian estimation of community size and overlap from random subsamples
Source: PLoS Comput Biol. 2022 Sep 19;18(9):e1010451. doi: 10.1371/journal.pcbi.1010451 (PMC9522272; doi:10.1371/journal.pcbi.1010451)
Supplement: Attachment — Submitted filename: Revisions - Johson & Larremore - Bayesian - PLOS Comp Biol.pdf [file pcbi.1010451.s003.pdf]

Dear Editors & Reviewers,

We are pleased to resubmit our manuscript for reconsideration after revisions. The manuscript has been renamed, “Bayesian estimation of ~~population~~ community size and overlap from random subsamples,” a change which reflects the suggestions of Reviewer 2 in particular.

In the resubmission, changes have been marked in blue text to facilitate review and readability. In addition to smaller changes to the paper, detailed below,

1. We have now published, documented, and tested the open-source GitHub repository that accompanies the manuscript. It includes the inference code, an installation guide, example code, and the raw data used to generate the manuscript’s figures.

2. We rewrote much of the introductory text concerning the descriptions of *P. falciparum*’s *var* genes.

3. We expanded our discussion of the limitations of the methods of this paper, and compressed the derivation in Appendix 2.

We hope that you will find the revised manuscript improved, and thank you for your continued consideration. We would also like to thank both reviewers and editors for their understanding in the longer-than-usual revision time, and apologize for this inconvenience.

Sincerely,

Daniel Larremore

Dan Larremore & Erik Johnson

Reviewer #1:

This study looks at the issue of estimating the overlap between two sets when only partial samples are available from each, and - unlike previous work (Larremore 2019) - when the true repertoire sizes of both sets are unknown. The authors take a statistical modelling approach, starting by constructing a generative model and then describing how this can be used to calculate likelihoods and perform Bayesian inference. The final method is demonstrated to have nice properties, including reducing bias in cases where there is sufficient data to overwhelm the prior, and of course having a measure of uncertainty that is missing from traditional point estimates.

Overall I think this is a strong paper. The method is very clearly described, and the general gist of the method could be followed even by those without a strong statistical background. There are clear advantages to the Bayesian formulation of the problem, and the extension presented here opens the previously described method up to a wider class of problems. The general nature of this problem also means it could find application in many areas.

We thank the reviewer for this encouraging assessment!

Other than some very minor points (see below), my only major criticism is around code availability. It is stated in the paper that an open-source implementation of the methods is freely available, and we're pointed to the code availability statement, which points us to a GitHub repository, which appears to be basically empty (I could just see a LICENSE, an empty README and a .gitignore at time of review). Given the great effort the authors have gone to to ensure there are no unnecessary mathematical obstacles to the user it seems a shame not to have a nicely packaged tool that can be used out of the box.

We apologize for this major oversight at submission. Our resubmission includes a GitHub repository with requirements, installation instructions, example code, and a version-minted DOI for access into the future.

There is some pretty heavy re-use of material from the earlier 2019 paper (e.g. the P. falciparum section). I didn't calculate the overlap between these two sets of text, but in some cases I suspect it's upwards of 90%, depending on the estimation method used. I leave it to the editor to decide how big of a problem this is for this particular journal.

We have rewritten all sections of the manuscript that re-used prior text.

My recommendation is to accept with minor changes, which include the points below and also tidying up the code availability.

Minor points:

1. In the description immediately before formula (A6), consider using j to index the second series (i.e. “let f_j be the number of times u_j appears in C”). Otherwise it gives the sense that f_i somehow corresponds to c_i, when in fact these are different indices.

Great suggestion. Fixed.

2. Typo: In formula (A7) a central dot is used as shorthand for multiplication, and also to mean the sequence of all values from c_2 to c_n. I think the latter should be a centre-justified ellipsis.

Agreed. Fixed.

3. The derivation of the formula p(R | n), i.e. using just the number of unique groups and not complete counts, is mathematically correct but overly complicated and probably unnecessary. The distribution of unique items in a multinomial sample is a reasonably standard expression that has been described before, see for example (https://arxiv.org/abs/1602.05822), section “The distribution of unique items”. Citing this common result would simplify the derivation here and avoid accidentally taking credit for past work.

We have used this suggestion to dramatically simplify the derivation, and cite the suggested paper and section.

4. In the “Inference” section, there is the statement “The true value of R is always contained within the inferred distributions”. This might be picky, but I don’t really like this statement. It’s not clear what “contained within” really means here, as even something in the 99.99th centile is contained within the distribution. Something along the lines of “confidence increases with increasing m” seems more appropriate.

We agree. After considering the value of the sentence in question, we decided to strike it entirely, as it is redundant to the previous sentence and the points in the paragraph that follows.

5. Grammatical mistake in the line “By drawing inferences both from this work also aligns with past efforts “.

Fixed.

Code availability points to a GitHub repository, which is empty at time of review and hence the code is not made available with the paper. This may be a simple mistake, for example updating the repository on a local branch but failing to push to main.

We have corrected this oversight.

Reviewer #2:

Johnson & Larremore proposed a new Bayesian estimation of beta-diversity that jointly estimates population sizes and the overlap. They showed that the estimates are unbiased when sampling efforts are large enough compared to the population sizes. This is an extension of Larremore (2019) PloS Comp Bio where population sizes are a fixed known quantity.

In general, I think the paper is clearly written, including the results. I still have several questions though regarding the definition and the scope of application.

We appreciate this encouraging feedback.

1) It seems that population size and repertoire size are used interchangeably in the article when describing R. However, what R really refers to is not population size, but the number of species in the population (as defined in the introduction). In ecology and evolution, population size has a very different meaning—usually in the order of 10^4-10^7. Clearly, in this article, R really refers to different species/genes per population. I think the authors should be consistent in their definition of R, and make it relevant to ecologists.

We have audited our use of the word *population* throughout the paper.

2) It would be nice if the proposed measure can be applied with data from published papers to check how SD_bayesian performs, and whether it changes any conclusions from the studies. It would be better if it's applied to ecological data where beta-diversity is calculated.

While we agree in principle, in practice this is made difficult by the fact that our approach requires both (1) abundance measurements—or at least presence-absence measurements *and* total sampling effort; and (2) an underlying or true uniform abundance, as in the sampling of genomic *var* genes where each is approximately equally likely to be sampled via bulk sequencing. Unfortunately, prior *var* papers report only presence-absence, and most applied ecology contexts strongly violate the uniform probability of sampling assumption. We would be interested in the Reviewer's thoughts if particular studies meeting these criteria (with available data) come to mind. Two *unpublished* studies that we are aware of meet these criteria, but we are unable to analyze their data.

3) On page 8, the authors stated that "In real data from P. falciparum , repertoires (and thus repertoire overlaps) larger than 60 are rarely observed [28, 15], decreasing the potential impact of this issue". However, many patients contain multiple infections of parasites, where the total isolate size is much larger than 60. It is worthwhile to discuss how that would influence the overlap estimation when the number of infections per isolate is unknown.

This is a great point. We have made two changes to address it. (1) We altered the Results section in question to now be clear about the fact that this method addresses overlap between parasites, not infections. (2) We treat this issue in a new limitations paragraph in the Discussion.

Aside from changes to the manuscript, we are unsure of how one would proceed *even if* MOI were known. For instance, if MOI=2 in an infection, it's unclear whether one would expect a total repertoire of ~120 (two totally unrelated infections) or ~60 (two highly related repertoires with nevertheless different haplotype barcodes). These are extreme cases contrived for discussion, but where, between them, any particular MOI>1 sample falls, would be unknown. This work will have to be left for the future.

4) R = Ra+Rb, and m = ma+mb, or m = ma=mb? It is not clear to me from the manuscript, how the difference between Ra, Rb or ma/mb would influence the results.

We have audited our used of *m* throughout the manuscript and added context in many places. We now more clearly note that *m_a* = *m_b* = *m* in many places and captions, and removed references to *m* in places where its inclusion was ambiguous or confusing. In the main text where we focus on a single population, we now explicitly note to the reader that subscripts will be dropped because there is only one population under consideration.

Have the authors made all data and (if applicable) computational code underlying the findings in their manuscript fully available?

The link to the github repo does not contain any codes yet.

We apologize for this oversight, and have fixed the issue. The repository is now complete with requirements README, installation instructions, and example code. We have also minted a version to provide better longevity, and the repository includes the data used to create all manuscript figures, in addition to the inference code itself.
